# Targeted rotavirus vaccination of high-risk infants; a low cost and highly cost-effective alternative to universal vaccination

**DOI:** 10.1186/1741-7015-11-112

**Published:** 2013-04-26

**Authors:** Patricia Bruijning-Verhagen, Marie-Josée J Mangen, Mariet Felderhof, Nico G Hartwig, Marlies van Houten, Léon Winkel, Wouter J de Waal, Marc JM Bonten

**Affiliations:** 1 Julius Center for Health Sciences and Primary Care, University Medical Center Utrecht, Huispostnummer STR.6.131, Postbus 85500, Utrecht 3508 GA, The Netherlands; 2 Department of Pediatrics, Spaarne Hospital, Postbus 770, Hoofddorp 2130 AT, The Netherlands; 3 Department of Pediatrics, Sint-Franciscus Hospital, Postbus 10900, Rotterdam 3004 BA, The Netherlands; 4 Department of Pediatrics, Kennemer Hospital, Postbus 417, Haarlem 2000 AK, The Netherlands; 5 Department of Pediatrics, Diakonessen Hospital, Postbus 80250, Utrecht 3508 TG, The Netherlands

**Keywords:** Rotavirus vaccination, Cost-effectiveness, Cost-utility, High-risk population, Targeted prevention, Hospitalization, Nosocomial infection, Mortality

## Abstract

**Background:**

The cost-effectiveness of universal rotavirus (RV) vaccination is controversial in developed countries. As a result, RV vaccination programs do not currently exist in most European countries. Hospitalization is the main driver of RV disease costs, and prematurity, low birth weight (LBW) and underlying medical conditions have been associated with RV hospitalization and complications. We investigated the cost-effectiveness of targeted RV vaccination of high-risk infants and universal RV vaccination versus no vaccination.

**Methods:**

Disease burden, mortality and healthcare costs of RV hospitalization for children with and without prematurity, LBW and congenital pathology were quantified in two hospital-based observational studies in the Netherlands. Cost-effectiveness analysis was based on an age-structured stochastic multi-cohort model of the Dutch population comparing universal RV vaccination and targeted vaccination of high-risk infants to no vaccination. The primary endpoint was the incremental cost-effectiveness ratio (ICER), with a threshold of €35,000/quality-adjusted life year (QALY) from the healthcare provider perspective. Sensitivity analyses included vaccine price and coverage, herd-immunity and QALY losses.

**Results:**

A total of 936 children with RV infection were included. Prematurity, LBW and congenital pathology were associated with increased risks of RV hospitalization (relative risks (RR) ranging from 1.6 to 4.4), ICU admission (RR ranging from 4.2 to 7.9), prolonged hospital stay (1.5 to 3.0 excess days) and higher healthcare costs (€648 to €1,533 excess costs). Seven children succumbed due to RV complications, all belonging to the high-risk population. Targeted RV vaccination was highly cost-effective and potentially cost-saving from the healthcare provider perspective with ICERs below €20,000/QALY in all scenarios with total (undiscounted) annual healthcare costs between -€0.1 and €0.5 million/year. Results were most sensitive to mortality rates, but targeted vaccination remained highly cost-effective up to reductions of 90% compared to observed mortality. Universal RV vaccination was not considered cost-effective (mean ICER: €60,200/QALY) unless herd-immunity and caretaker QALY losses were included and vaccine prices were €60 at most (mean ICER: €21,309/QALY).

**Conclusion:**

We recommend targeted RV vaccination for high-risk infants in developed countries.

## Background

Rotavirus (RV) vaccination reduces severe RV gastroenteritis (GE), associated healthcare utilization and mortality among young children [1,2]. Universal infant vaccination with either the monovalent life-attenuated vaccine (RV1) or the pentavalent human-bovine reassortant vaccine (RV5) has, therefore, been recommended by professional healthcare organizations worldwide [3-7] but results of cost-effectiveness analyses of RV vaccination in developed countries yielded conflicting results [8-11]. Consequently, RV vaccination programs have been introduced in only a very limited number of European countries [12].

Although RV is widely considered a universal pediatric infection with young age as the only important risk factor, in the developed world hospitalization due to RV, the main driver of RV associated healthcare costs, seems to be associated with underlying chronic disease, congenital disorders, prematurity and low-birth weight (LBW) [13-24]. Moreover, these children are more prone to complicated RV disease courses with more frequent ICU admission [18]. RV infection in premature or LBW infants has been associated with necrotizing enterocolitis, encephalopathy [25-29] and increased diarrheal mortality [30-32].

A targeted vaccination strategy for RV, in which vaccination is offered to high-risk infants only, has not been economically evaluated thus far. We, therefore, set out to determine the cost-effectiveness of such an approach in the Netherlands, where universal RV vaccination has not been implemented yet, similar to the situation in most European countries. We first quantified RV related hospitalizations in the Netherlands and identified patient groups at increased risk of RV hospitalization or with increased healthcare needs when hospitalized. Subsequently, these data were supplemented with relevant available epidemiological data to determine cost-effectiveness of both universal and targeted RV vaccination strategies compared to no vaccination from a healthcare provider perspective.

## Methods

### Rotavirus hospitalizations; observational study

The methodology of this study has been described elsewhere [33]. In brief, laboratory confirmed RV related pediatric hospitalizations occurring in four participating hospitals (three general hospitals, one tertiary care center) during a five-year period (December 2005 to November 2010) were retrospectively studied. RV underreporting was subsequently assessed by hospital-based active surveillance during the 2011 RV season in the same four hospitals [33].

A comprehensive chart review was performed for each case extracting data on RV disease course, healthcare resource utilization and patient’s medical history to identify conditions potentially associated with increased clinical vulnerability, such as prematurity of <36 weeks gestational age and/or LBW (<2,500 grams) and complex chronic conditions. Complex chronic conditions were those that (1) are expected to last longer than 12 months and (2) involve either several different organ systems or one organ system severely enough to require specialty pediatric care and hospitalization. This classification characterizes a group of patients with increased healthcare needs and mortality [34-36]. We further classified complex chronic conditions into those with a congenital origin (that is, severe congenital pathology) and those with onset later in life.

Prevalence rates for prematurity/LBW and severe congenital pathology were also derived for the Dutch infant population from national disease and birth registries, covering 96% of the Dutch infant population [37,38]. In addition, a nested case–control study was performed to investigate if the same conditions increased the risk of nosocomial RVGE compared to otherwise healthy age-matched hospital controls [see details in Additional file 1].

Healthcare resource utilization, assessed at the individual patient level, was used for cost calculations, adapting standard cost prices and charges [see Additional file 1: Table S1] [39,40]. Costs included hospitalization days, preceding emergency department visits, contact isolation precautions [41] and ambulance transportation. For nosocomial RVGE costs for RV related excess hospitalization days were used [33], or, when hospitalization was not prolonged, isolation costs and RVGE related diagnostic and therapeutic costs. This study was approved by institutional review board of the University Medical Centre Utrecht.

### Statistical analysis

The prevalence of prematurity/LBW and severe congenital pathology among RV hospitalizations compared to the general infant population were used to compute Risk Ratios (RR). To account for the clustered study design and for oversampling of tertiary-care hospitalizations compared to their national share in pediatric hospitalizations (20%), weighted prevalence estimates were calculated with variance estimated using Taylor series linearization [42].

Rates of ICU admission and RV-related deaths were compared between RV patients with and without potential high-risk conditions by computing RR. Length of stay or excess hospitalization days in the case of nosocomial RVGE and healthcare costs were compared by t-test, using the arithmetic mean despite the usually skewed distribution. The arithmetic mean is considered most informative in evaluations designed to have an impact on medical policy, because it is the total disease burden that is important [43].

Any of the assessed risk factors (prematurity/LBW or congenital pathology) that were associated with increased risk of RV hospitalization, nosocomial RVGE, RV-related death and/or increased length of stay were included to determine eligibility for targeted vaccination, excluding those who suffered from severe immunodeficiencies, in whom RV vaccination is contra-indicated [44].

Differential RV hospitalization rates were calculated for eligible and ineligible children from RVGE numbers adjusted for underreporting using weighted estimation [42]. Similarly, weighted mean hospitalization costs for community-acquired and nosocomial RV infections were calculated among eligible and ineligible children.

Analyses were performed using R software, version 1.14.1.

### Model design

We used an age-structured, discrete time-event, stochastic multi-cohort model of the Dutch population, as previously described by Mangen *et al*. [45] to investigate the cost-effectiveness of adding RV vaccination to the Dutch infant immunization program under two scenarios: (1) universal vaccination and (2) targeted vaccination of high-risk infants.

Strategies were compared assuming an annual birth cohort of 180,000 infants, equivalent to the 2010 Dutch birth cohort. The effect of vaccination was modeled as a reduction in RVGE and associated health outcomes in vaccinated compared to non-vaccinated children between 0 and 15 years old with RV disease risk stratified by age and time since vaccination [see Additional file 1: Figure S1]. Time steps of one month were used for ages 0 to 11 months and of one year thereafter. Effects were modeled over a time-horizon of 20 years with year one being the start of either vaccination program. The model was adapted to simulate targeted vaccination by splitting the population into a vaccination eligible and an ineligible fraction. We assumed no effect on adult RV infections from any of the infant vaccination strategies.

### Model parameters

Estimates of RV infection rates, outpatient healthcare visits and related direct and indirect healthcare costs for different age-groups were derived from existing epidemiological sources as previously described by Mangen *et al*. (Table 1) [45]. Hospitalization rates and costs for children eligible and ineligible for targeted vaccination and for combined groups were derived from our multi-center observational study.

**Table 1:** Parameters for model input

**Parameter**	**Value (95% CI)**			**Distribution**	**Data source**	**Method**
	**Total population**	**Ineligible population**	**Eligible population**			
**Birth cohort (%)**	182,662	168,215 (92.1%)	14,448 (7.9%)	-	Statistics Netherlands [46], Dutch Perinatal Registry [38], Eurocat [37]	Published results on birth cohort size and prevalence of high risk conditions
**RV incidence**						
<1 year	18,075 (11,768; 22,932)	Calculated	Calculated	Pert	Community-based cohort study [47]	Incidence based on simulations from original study data updated to 2011 population size, see Mangen *et al*. for details [45] Distribution among eligible and ineligible based on relative size of each group in birth cohort
1 to 4 years	42,218 (24,711; 56,272)					
5 to 64 years	147,997 (41,573;282,866)					
5 to 9 years	6.2% of 5 to 64 years					Based on age-distribution of cases 5 to 64 years in original study data
10 to 14 years	2.9% of 5–64 years					
GP visits 0 to 1 years	21.2% (12.8; 26.5)	Calculated	Calculated	Pert	GP based cohort study [48]	Percentage of all RV cases, based on simulations from original GP study data, see Mangen *et al*. for details [45]. Distribution among eligible and ineligible based on relative size of each group in birth cohort
GP visits 1 to 4 years	18.7% (16.4; 19.9)					
GP visits 5 to 14 years	4.0% (1.8; 4.7)					
Hospitalization (95% CI)	Calculated	3,884 (3,244; 4,524)	491 (357; 626)	Pert	RoHo-study	Weighted incidence estimation based on original study data, see Bruijning-Verhagen *et al.* for details [33].
Nosocomial (95% CI)	Calculated	227 (162; 293)	269 (172; 365)	Pert	RoHo-study	Weighted incidence estimation based on original study data, see Bruijning-Verhagen *et al*. for details [33]
Mortality rate (per 1,000 RV hospitalizations)	Calculated	0.00 (0.00; 0.04)	0 81 (0.36; 1.46)	Triangular	RoHo study, External dataset Sophia Children’s hospital	Observed mortality cases from both sources were combined for weighted mortality rate estimation
Age distribution of RV hospitalizations and fatal cases	Additional file 1: Table S2				RoHo study	
**Utilities RV gastroenteritis**	**QALY Loss**					
Mild (RV episode without medical care)	0.0011/0.002^a^				GP study in Canada [49], Previous CEA [9]	Published data
Moderate (GP visit)	0.0022/0.004^a^					
Severe (Hospitalization)	0.0022/0.004^a^					
Nosocomial	Calculated	Calculated	Calculated		RoHo Study	Based on severity distribution of nosocomial cases observed in RoHo-study
Mortality	Calculated	80.7 minus patient’s age	Simulated, whereby assuming a life expectancy of 1; 20; 41.3 minus patient’s age with probability of 1/3 each^b^	Uniform	Statistics Netherlands [46], Expert opinion	For ineligible: Based on average life expectancy in the Netherlands. For eligible: Based on expert panel†
**Direct healthcare costs (Euro)**						
Gastroenteritis episodes without medical care	0			Fixed		
Standard GP visits	29				Guidelines for health-economic evaluations [39]	Standard Cost Prices. See Mangen *et al*. for details [45]
Home visit GP	45					
GP consultation by phone	15					
Prescriptions	40				Community-based cohort study and GP based cohort study [49,50]	See Mangen *et al*. [45]
Laboratory costs	73					
Hospitalization	Calculated	2,179 (2,027;2,330)	2,550 (2,508; 3,606)	Pert	RoHo study	Weighted estimates from original study data, see Additional file 1
Nosocomial	Calculated	1,995 (1,242; 2,748)	2,129 (1,203; 3,055)			
**Direct non-healthcare costs**						
RV episode without medical care	Additional diapers			Uniform	Assumption	See Mangen *et al*. [45]
GP visits	Additional diapers and travel costs				Guidelines for health-economic evaluations [39]	
Hospitalization	Travel costs			Pert		
Nosocomial						
**Indirect non-healthcare costs**^**c**^						
Costs per hour work loss (euro)	31.11			Fixed	Statistics Netherlands [48] Guidelines for health-economic evaluations [39]	See Mangen *et al*. [45]
Hours of work loss for RV episode without medical care	0.93; 1.36; 0.84 for ages 0 to 4; 5 to 9 and 10 to 14 years respectively			Uniform	Community-based cohort study and GP based cohort study [49,50]	Dependent of patient-age. See Mangen *et al*. [45]
Hours of work loss GP visits	1.35; 1.98; 1.23 for ages 0 to 4; 5 to 9 and 10 to 14 years respectively			Uniform		
Hours of work loss Hospitalization	37.32				hospital based observational study [51]	Based on the findings from Friesema *et al*. [52] for children up to 18, Further details see Mangen *et al*. [45]
Hours of work loss Nosocomial	24.58					Based on the findings from Friesema *et al*. [52] for children up to 18, adjusted for excess duration of hospitalization among nosocomial in RoHo study (2.7 versus 2.9 days)
**Vaccine efficacy**	Table 2				Vaccine trials [53-57]	
**Herd-immunity**	**Universal RV vaccination**	**Targeted RV vaccination**				
	30% (0% to 46%)	-		Triangular	Observational studies from US [58,59], Australia [60,61], Belgium [62]	Published data
**Vaccination costs**						
RV1	60; 75; 90	80; 100; 120			Previous CEA [10] For Eligible: Assumption	Assumed tender Price
RV5	60; 75; 90	80; 100; 120				
Startup costs first year	218,440	-				
Application costs	6.44					See Mangen *et al*. [45]
Administration costs	1.64					

^a^includes QALY’s lost by caretakers; ^b^all observed fatal RV cases occurred among patients with severe congenital conditions associated with limited life-expectancy (LE). To generate valid estimates of life years lost (YLL) due to RV for each fatal case, four pediatricians (PB, MF, MvH, NH) were individually asked to provide estimates of LE without RV infection based on the patient’s medical records. Pediatricians were requested to estimate for each individual fatal RV case the probability that LE without RV would have been ≤1 year, 1 to 20, 20 to 40 years or comparable to healthy infants. Independent estimates for all seven observed cases were pooled to generate a distribution of mean YLL per fatal RV case; ^c^costs of third person taking care of sick child.

GP: general practitioner; QALY: quality-adjusted life years; RV: rotavirus.

Mortality due to RV was determined by combining data from the multi-center observational study and from another study in a Dutch tertiary-care hospital. In this study RV-related mortality was determined for all children who had died within three weeks of confirmed RV infection between 2000 and 2006. Conservative estimates were used for national mortality figures, assuming that fatal cases exclusively occurred among RV hospitalizations at tertiary-care centers without underreporting.

An expert panel of four pediatricians was consulted to determine years of life lost (YLL) accountable to RV infection among observed fatal cases (Table 1). This approach was used to take into account the reduced life expectancy in children with complex chronic conditions.

We used Quality Adjusted Life Years (QALYs), the product of the health-state utility and the length of time in that state, to weigh losses as a result of RV episodes requiring different levels of healthcare, similar to those used in previous cost-effectiveness analyses [9,10,45]. QALY losses due to RV mortality were based on YLL estimates for observed fatal RV cases.

European vaccine efficacy data were used for age-specific vaccination effects (Table 2) [53-56,63]. Linear waning immunity was assumed for the third, fourth and fifth year post-vaccination, and zero protection thereafter. Rotavirus genotype distribution in the Netherlands is comparable to the observed genotype distribution in European vaccine efficacy trials. Overall, G1P [8] is the dominant strain and G2P [4], G3P[8], G4P [8] and G9P [8] are common co-circulating strains with year-to-year variability in strain distribution [52]. We assumed 88% adherence to vaccination recommendations for both universal and targeted RV vaccination, the observed current vaccine coverage in neighboring Belgium where universal RV vaccination was implemented in 2007 [64], and used coverage rates from 65% to 97% in sensitivity analysis.

**Table 2:** Vaccine efficacy estimates against mild, moderate and severe RV gastroenteritis

	**Vaccine efficacy**^**a**^				**Method**	**Source**
**RV1**	**Mild (RV episode without medical care)**	**Moderate (GP visit)**	**Severe (Hospitalization)**^**b**^	**Nosocomial**^**c**^		
After first dose	Calculated	Calculated	89.8% (8.9 to 99.8)	Calculated	(Calculated from) Published data Efficacy for mild and moderate cases after first season calculated from efficacy ratios for mild, moderate, severe during first season	[53]
First season (after second dose)	71.7% (50.4 to 83.9)	91.8% (84.0 to 96.3)	100% (81.8 to 100)			
Second season	50.5% (24.3 to 67.7)	76.2% (63.0 to 85.0)	92.2% (65.6 to 99.1)			
Third-fifth season	Calculated	Calculated	Calculated		Efficacy during third to fifth season calculated as linear decline equal to reduction between first and second season	
**RV5**						
After first dose	Calculated	Calculated	88% (65 to 97)	Calculated	(Calculated from) Published data Efficacy after first dose and between first and second dose for mild and moderate cases calculated from efficacy ratios for mild, moderate, severe during first season	[57]
After second dose	Calculated	Calculated	88% (69 to 96)			
First season (after third dose)	65.1% (54.1 to 73.5)	72.0% (63.2 to 78.9)	94.8% (89.4 to 97.8)		(Calculated from) Published data	[54,55]
Second season	49.8% (27.0 to 65.4)	58.5% (40.1 to 71.7)	90.8% (76.9 to 97.1)			
Third season	Calculated	Calculated	100.0% (27.9 to 100)		Efficacy during third season for mild and moderate cases calculated from efficacy ratios for mild, moderate, severe during second season	[55]
Fourth-fifth season	Calculated	Calculated	Calculated		Efficacy during third to fifth season calculated as linear decline equal to reduction between first and second season	

^a^Vaccine efficacy was assumed equal between eligible and ineligible, data were modeled as Pert distribution; ^b^efficacy against fatal RVGE was assumed equal to efficacy for severe disease; ^c^efficacy against nosocomial infection based on severity distribution in original study data.

GE: gastroenteritis; GP: general practitioner; RV: rotavirus.

Vaccine costs for universal RV vaccination were based on Rozenbaum *et al*. who assumed that tender processes lower vaccine prices by almost 50% (€75 per vaccine course) compared to the current free market price [10]. Targeted vaccination was assumed to generate price reductions of 25% (€100 per vaccine course). We also included scenarios with the free market price for both vaccination strategies. We assumed vaccine doses would be administered during routine immunization clinic visits at a standard application fee of €6.44 per vaccination [45].

Indirect vaccination effects (herd-immunity) among unvaccinated children were considered as part of the sensitivity analysis (Table 1) [58-60]. No herd-immunity was assumed in the case of targeted vaccination, as vaccine coverage was considered too low for herd-immunity to occur.

### Cost-effectiveness analysis

Our primary perspective was that of the healthcare provider and a societal perspective was included in sensitivity analysis taking non-healthcare costs into account, updated from Mangen *et al*. with additional data on parental work loss due to RV hospitalizations in children [65]. All costs were converted to 2011 Euros. A 3% discount rate for costs and benefits was used in base-case scenarios according to World Health Organization (WHO) guidelines [66]. Other discount rates, including those recommended for Dutch health economic evaluations, were used in sensitivity analysis [39]. Although there is no consensus on a cut-off point for good value for resources, we present our results in the context of commonly cited thresholds per QALY of $50,000 equivalent to €35,000 [67,68]. This amount is approximately equal to the Dutch Gross Domestic Product per capita in 2011, the recommended threshold for highly cost-effective interventions by the WHO [66]. In addition, we used the unofficial threshold of €20,000/QALY commonly applied in the Netherlands for preventive healthcare interventions.

Total net healthcare costs for either vaccination strategy compared to no vaccination are reported (cost-analysis) as well as incremental cost-effectiveness ratios (ICERs), representing costs per QALY gained comparing either strategy with no vaccination. Strategies were considered cost-effective if they generated ICER’s less than a willingness-to-pay threshold of €35,000/QALY from the healthcare provider perspective. An ICER below €20,000/QALY was considered highly cost-effective. As additional scenario analysis, we also calculated incremental costs and QALY’s gained for universal RV vaccination compared to targeted vaccination.

The simulation model was built in Microsoft Excel using add-in software @Risk, version 5.5 (Palisade). Results are presented as means and 95% confidence interval (CI) of simulated results, based on 10,000 iterations. Parameters were varied simultaneously in probabilistic sensitivity analyses, performing random draws from distributions. Distributions were chosen based on parameter characteristics and level of certainty. Input parameters and their distributions with corresponding information source are presented in Table 1. In addition, we performed one-way sensitivity analysis to determine variables which were most influential on model results.

## Results

### Rotavirus hospitalizations; observational study

Overall, 944 RV infections were identified. After excluding six patients with asymptomatic disease and two without medical records available, 936 patients were analyzed. RVGE was community-acquired and nosocomial in 770 (81%) and 176 (19%) episodes, respectively [see Additional file 1: Table S4].

In 134 patients (14%) RVGE occurred before 15 weeks of age and would not be prevented by vaccination, unless by herd-immunity. Prevalence of prematurity <36 weeks was 9% (n = 83), of low birth weight was 11% (n = 104) and of complex chronic conditions at the time of RV infection was 23% (n = 219). The latter was more frequent among nosocomial than among community-acquired infections (64% versus 14%, *P* <0.0001). Most of these patients (n = 116, 53%) suffered from severe congenital pathology. Based on weighted prevalence of prematurity, LBW and congenital pathology among RV hospitalizations and the general infant population, all three conditions were significantly more common among children hospitalized for RVGE and were, therefore, classified as high-risk for RV hospitalization (mean RR: 1.7; 1.6; 4.4, respectively; Table 3). RVGE-related ICU admission occurred more frequently among children with prematurity, LBW and congenital pathology than among otherwise healthy patients (mean RR ranging from 4.2 to 7.9). Mean length of stay was increased by 1.5 to 3.0 days and mean healthcare costs were €648 to €1,533 per patient higher (Table 3). Results from the nested matched case–control study demonstrated increased risks of acquiring nosocomial RVGE for prematurity (aOR: 3.3, 95% CI: 1.5 to 7.3), LBW (aOR: 3.2, 95% CI: 1.5 to 7.1) and congenital pathology (aOR: 3.6, 95% CI: 1.8 to 7.0) compared to healthy hospitalized controls (details in Additional file 1).

**Table 3:** Prevalence of high risk conditions among RV hospitalizations and their association with disease outcome and healthcare utilization

	**Prevalence**						***P*-value**^**b**^	**RR**^**b**^
	** Observational study**		**Weighted estimates national RV hospitalizations (95% CI)**		**General infant population**^**a**^			
**High Risk Conditions**	**%**	**N**	**%**	**N**	**%**	**N**		
GA < 36 weeks	8.9	83	6.8 (5.1; 8.5)	347 ( 243; 451)	4.3	7617	0.005	**1.7 (1.2; 2.8)**
LBW	11.1	104	8.8 (6.6; 11.1)	462 (309; 615)	6.0	10545	0.014	**1.6 (1.1; 2.3)**
Congenital pathology	12.4	116	6.2 (4.9; 7.4)	309 (244; 374)	1.5	2719	<0.0001	**4.4 (3.4; 5.4)**
	**Healthy (N = 657)**		**High risk conditions**					
			**GA <36 weeks (N = 83)**		**LBW (N = 104)**		**Congenital pathology (N = 116)**	
**Outcome and healthcare utilization**	**N (%)**		**N (%)**	**RR (95% CI)**	**N (%)**	**RR (95% CI)**	**N (%)**	**RR (95% CI)**
ICU admission	4 (0.6%)		4 (4.8%)	**7.9 (2.0; 31.1)**	3 (2.9%)	**4.7 (1.1; 20.9)**	3 (2.6%)	**4.2 (1.0; 18.7)**
RV related death (number,%)	0		0		0		2 (1.7%)	**NA**
				**Mean difference (95% CI)**		**Mean difference (95% CI)**		**Mean difference (95% CI)**
LOS (mean, SD)	3.6 (2.1)		5.2 (4.7)	**+1.6 (0.1; 3.0)**	5.1 (4.5)	**+1.5 (0.3; 2.7)**	6.6 (4.2)	**+3.0 (1.9; 4.1)**
Healthcare costs (mean, SD)	2,203 (2,113)		3,001 (3,407)	**+798 (28; 1,568)**	2,851 (3,206)	**+648 (−2; 1,297)**	3,737 (3,500)	**+1,533 (867; 2,199)**

^a^Dutch birth cohort alive after one month, 2005-2008^38^; ^b^Comparing weighted RV hospitalizations prevalence to population prevalence.

GA: gestational age; LBW: low birth weight; LOS, length of stay; RR, relative risk; RV, rotavirus; SD: standard deviation.

The figures in bold indicate the results of calculations form other columns in the table and represent the main findings.

### RV mortality

Two RV related fatalities were observed in the multi-center study and an additional five among 214 confirmed RVGE episodes over six years in the second observational study at a different tertiary care center. All seven had congenital pathology and two patients also had a history of LBW. One child died before 2 months of age, the remaining six children died between 2 and 14 months of age.

### RV epidemiology and vaccination effects

Without vaccination, there were an estimated 75,000 (95% CI: 58,000 to 90,000) RVGE episodes annually, with 4,870 (95% CI: 4,310 to 5,430) hospitalizations including 500 nosocomial infections among children 0 to 15 years old, generating €11.9 (95% CI: 10.5 to 13.3) million in total healthcare costs and €18.2 million (95% CI: 16.2 to 20.3) when societal costs are included (Table 4). An estimated 6.5 children (95% CI: 3 to 11) die prematurely due to RV each year in the Netherlands. A total of 257 QALY’s (95% CI: 136 to 422) are lost due to RV, of which 170 (95% CI: 50 to 330) are due to fatal RV cases.

**Table 4:** Annual results of universal and targeted RV vaccination compared to no vaccination under base-case assumptions

	**RV disease burden (95% CI)**^**a**^				** RV disease costs**^**a **^**(€ million)**		**Vaccination costs (€ million)**
	**Disease episodes (x1000)**	**Hospitalizations**^**b**^	**Fatal cases**	**QALY’s lost (undiscounted)**	**Direct healthcare costs (undiscounted)**	**Societal costs (undiscounted)**	**(undiscounted)**
**No vaccination**	74.1(57.8; 90.0)	4,870 (4,310; 5,430)	6.5 (3.2; 11.0)	257 (136; 422)	11.9 (10.5; 13.3)	18.2 (16.2; 20.3)	-
**Targeted RV vaccination**							
RV1	67.3 (51.3; 82.4)	4,370 (3,890; 4,870)	0.7(0.2; 1.6)	119 (79; 177)	10.5 (9.3; 11.8)	16.4 (14.6; 18.2)	1.5
Percent reduction	8%	10%	89%	54%	12%	10%	
RV5	67.4 (51.5; 82.7)	4,384 (3,892; 4,870)	0.8 (0.3; 1.7)	121 (80; 184)	10.6 (9.4; 11.8)	16.4 (14.6; 18.2)	1.6
Percent reduction	8%	10%	88%	53%	11%	10%	
**Universal RV vaccination**							
RV1	40.6 (30.1; 51.2)	1,370 (1,150; 1,650)	0.4 (0.2; 0.8)	60 (42; 81)	3.4 (2 8; 4 1)	5.9 (5.0; 6.9)	15.2
Percent reduction	45%	72%	94%	77%	71%	67%	
RV5	42.6 (31.7; 53.6)	1,440 (1,210; 1,710)	0.5(0.2; 0.9)	66 (45; 91)	3.6 (3.1; 4.3)	6.3 (5.3; 7.3)	16.7
Percent reduction	43%	70%	92%	75%	70%	65%	

^a^Results reflect those five years and more after initial implementation of a vaccination strategy when steady state is reached; ^b^including nosocomial infections. CI:confidence interval; QALY:quality-adjusted life year; RV: rotavirus.

A universal RV vaccination program (at €75 per vaccine course per child) generates €15.2 million in healthcare costs annually when the two-dosage RV1 and €16.7 million when the three-dosage RV5 is used. The difference is explained by the additional application costs of a third RV5 dose. A total of 3,500 (95% CI: 3,050 to 3,960) and 3,430 (95% CI: 2,980 to 3,880) RV hospitalizations are avoided by RV1 and RV5, respectively. QALY’s gained mount to 194 (95% CI: 83 to 348) for RV1 and 188 (95% CI: 80 to 342) for RV5. On average, 6.1 (95% CI: 3.0 to 10.2) fatal cases are avoided.

Using targeted vaccination, approximately 8% of the infant population would be eligible for vaccination (that is, children with one or more high-risk conditions). At €100 per vaccinated child, annual vaccination costs are €1.5 million or €1.6 million (RV1 or RV5). Five hundred (RV1, 95% CI: 420 to 590) or 490 (RV5, 95% CI: 410 to 570) hospitalizations are avoided while the number of avoided fatal infections is similar to results for universal RV vaccination (5.8 cases, 95% CI: 3.0 to 9.5). A total of 137 QALY’s are gained.

As outcome results for either vaccine were almost identical, further analyses presented are based on RV1 vaccination with results for RV5 available in Additional file 1: Table S5.

**Figure 1:**
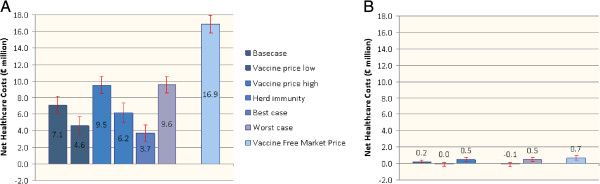
**Comparison of annual net healthcare costs for RV vaccination strategies.** Net undiscounted annual healthcare costs for universal (**A**) and targeted RV vaccination (**B**) compared to no vaccination under different assumptions and corresponding 95% CI. ‘Basecase’ represents results when the vaccine price per course is €75 per child, coverage is 88%, and no herd immunity is present. ‘Vaccine price low’ and ‘high’ represent results for a vaccine price per course of €60 and €95 per vaccinated child, respectively. ‘Herd immunity’ includes protection of unvaccinated children. A scenario with herd immunity effects was not included for targeted vaccination. ‘Best case’ represents results from a low vaccine price, coverage of 97% and presence of herd immunity. ‘Worst case’ represents a high vaccine price, coverage of 65% and no herd immunity. ‘Vaccine Free Market Price’ shows results when the current listed vaccine price is used without any tender effects. CI:confidence interval; RV: rotavirus.

Net healthcare costs (defined as vaccination costs minus healthcare savings) were compared for different scenarios (Figure 1). Undiscounted annual net healthcare costs of universal RV vaccination compared to no vaccination varied between €3.7 and €9.6 million, depending on assumptions about vaccine price, herd-immunity and vaccine coverage. Results of targeted vaccination versus no vaccination varied between net savings of €0.1 million up to maximum costs of €0.5 million. Comparing the free market vaccine price in both strategies resulted in a difference of €16 million annually (€16.9 versus €0.7 million for universal and targeted vaccination, respectively).

**Figure 2:**
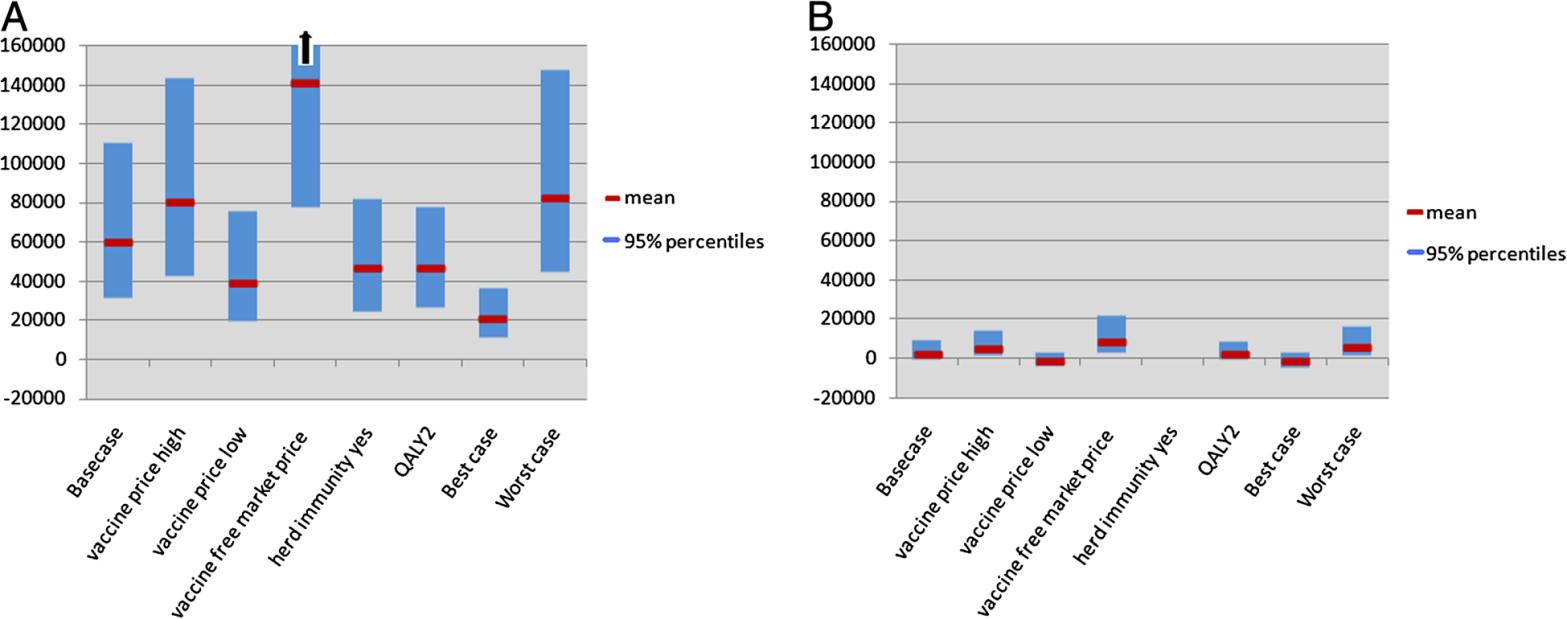
**Comparison of cost-effectiveness of RV vaccination strategies.** Cost per QALY gained (mean and 95% CI) for universal (**A**) and targeted RV vaccination (**B**), using a healthcare provider perspective and a discount rate of 3% for both costs and effects, under different assumptions. ‘Basecase’ represents results when the vaccine price per course is €75 per child, coverage is 88%, and no herd immunity is present. ‘Vaccine price low’ and ‘high’ represent results for vaccine price per course of €60 and €95 per vaccinated child, respectively, for universal RV vaccination and vaccine price per course of €80 and €120 for targeted RV vaccination. ‘Herd immunity’ includes protection of unvaccinated children (only used in universal RV vaccination). ‘QALY2’ represents results when QALY loss of caretakers is taken into account. ‘Best case’ represents results from a low vaccine price, coverage of 97%, including caretaker QALY’s and presence of herd immunity. ‘Worst case’ represents a high vaccine price, coverage of 65%, no caretaker QALY’s included and no herd immunity. ‘Vaccine Free Market Price’ shows results when the current listed vaccine price is used without any tender effects under base-case assumptions. CI: confidence interval; QALY: quality-adjested life year; RV: rotavirus.

Universal RV vaccination compared to no vaccination is unlikely to be cost-effective from the healthcare provider perspective at a willingness-to-pay threshold of €35,000/QALY gained (Figure 2 and Additional file 1: Figure S2). Among the different scenarios analyzed for universal RV vaccination, the only cost-effective scenario included both herd immunity and caretaker QALY losses and assumed a vaccine price of €60/child (ICER: €21,309/QALY, 95% CI: 11,079 to 36,047). Targeted RV vaccination is highly cost-effective in all scenarios tested. The best case scenario is dominant with net discounted mean savings of €60,000 and 67 QALY’s gained compared to no vaccination. The maximum ICER for targeted vaccination is €8,700/QALY when using the free market price (Figure 2). Moving from targeted vaccination to universal RV vaccination under base-case assumptions generates €5.3 million in mean incremental costs and 33 additional QALY’s gained. The mean ICER for universal versus targeted vaccination is €162,000/QALY using a healthcare provider perspective (Table 5). Outcome results from the societal perspective and analyses based on different discount rates are provided in Additional file 1: Table S6.

**Table 5:** Mean costs per different health outcome comparing different RV vaccination strategies under base-case assumptions (RV1) using a healthcare provider perspective

	**Targeted versus no vaccination**	**Universal versus no vaccination**	**Universal versus targeted vaccination**
**Costs (€)**^**a**^			
per case avoided	21	174	191
per life year saved	2,400	96,600	894,000
per fatal case	0.03^b^	1.03^b^	21.60^b^
per QALY gained	2,600	60,200	162,000

^a^A discount rate of 3% was used for both costs and effects; ^b^€million. QALY, quality-adjusted life year; RV, rotavirus.

**Figure 3:**
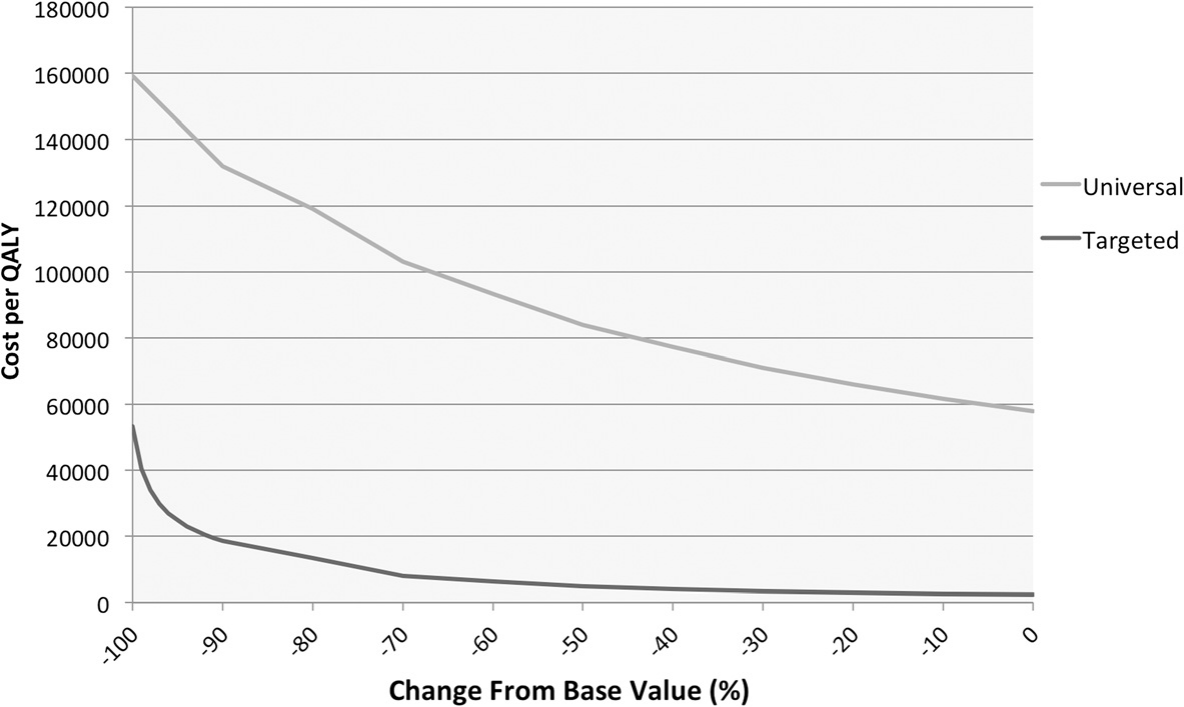
**Mean cost per QALY gained by universal (grey line) and targeted (black line) RV vaccination, using a healthcare provider perspective and a discount rate of 3% for both costs and effects, as a function of change in mortality rate between 0% and −100% (that is, no mortality at all) compared to baseline.** QALY: quality-adjusted life year; RV: rotavirus.

Cost-effectiveness was most sensitive to the estimated mortality rate. We, therefore, included a threshold analysis to identify the cut-off value of the parameter at which targeted vaccination would no longer be cost-effective. Targeted vaccination remained highly cost-effective up to a reduction of 90% in mortality rate, which translates to less than 1 RV death per year (Figure 3).

**Figure 4:**
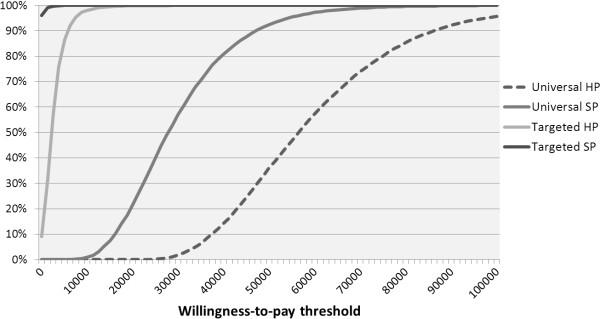
**Probability of willingness-to-pay at different thresholds for universal and targeted RV vaccination versus no vaccination under base-case assumptions showing results for both healthcare provider (HP) and societal perspective (SP).** RV: rotavirus.

Choosing between competing strategies requires consideration of cost-effectiveness acceptability curves, which visualize the probability of cost-effectiveness dependent on the willingness-to-pay for health benefits. At a threshold of €35,000/QALY, we estimate the probability of cost-effectiveness of universal RV vaccination under base-case assumptions from the healthcare provider perspective to be 6%, and 71% from a societal perspective (Figure 4). The probability of cost-effectiveness of targeted vaccination both at the €35,000/QALY and €20,000/QALY thresholds is 100% for both healthcare provider and societal perspectives.

## Discussion

Based on detailed Dutch epidemiological data analyzed in an age-structured, discrete-time event, stochastic multi-cohort model, we conclude that targeted RV vaccination of high-risk infants is highly cost-effective and potentially cost-saving in the Netherlands. Cost-effectiveness estimates were most sensitive to RV mortality rates, but targeted RV vaccination remained cost-effective when mortality would be 90% lower than observed.

In our analysis, universal RV vaccination was not considered cost-effective from the healthcare provider perspective and would only become cost-effective when herd-immunity and caretaker QALY losses were included and if vaccine prices would be at most €60/child. These results are in line with results from other European cost-effectiveness analyses that have used comparable methodology and QALY loss estimates for RVGE. Universal RV vaccination was not considered cost-effective from the healthcare provider perspective in Belgium, England and Wales, France, the Netherlands and Ireland, and was cost-effective in Finland only [9,69]. Our analysis demonstrated that universal RV vaccination could, however, be considered cost-effective from the societal perspective at the €35,000/QALY threshold. These findings differ somewhat from previous economic analyses of universal RV vaccination in the Netherlands [9,10,45] which can be explained by the updated and more reliable parameter estimates used. Incidence and costs of RV hospitalizations in the Netherlands determined in our study are comparable to estimates from Germany, Finland and the UK and another recent Dutch observational study [65,70-72]. Previously, lower incidence and cost estimates were derived for the Netherlands by using indirect methods combining sentinel laboratory data and hospital discharge codes [73,74]. The accuracy and completeness of methods using discharge codes has been criticized and depends on local coding practices [75-77]. In addition, we could include recent estimates of parental work loss in children hospitalized for RV [51].

Our analysis did not account for potential costs and QALY losses associated with vaccination induced intussusception. Based on observed intussusception risks attributable to RV vaccination in different populations, 0 to 9 additional cases would occur each year in the Netherlands when universal RV vaccination is implemented [78-86]. Clearly, this could have a negative impact on cost-effectiveness, although overall effects may be small. Furthermore, the recent reports on an increased risk of intussusception after the first dose of RV vaccine may raise concerns about exposing healthy children at low risk of RV-related complications to vaccination risks [87,88].

Our study confirms that prematurity, LBW and congenital pathology are important risk factors for RV hospitalization and increased healthcare needs. Furthermore, we observed RV mortality exclusively among patients with any of these high-risk conditions. Although absolute numbers were low, similar observations in other European and US studies and the association between diarrhea-related mortality and birth weight confirm the existence of differential mortality risks [17,19,30-32]. Of note, in five out of seven patients who succumbed the underlying illness rather than RV was stated as the cause of death in death-records. Yet, in these patients RV caused a profound medical deterioration leading to premature death, as confirmed by expert review of case histories. These findings suggest that among children with severe underlying conditions fatal RV disease is underreported.

Although limited data are available on vaccine safety and efficacy among high risk patients, protection provided by RV vaccine was comparable in premature and non-premature infants without additional safety risk [89-91]. Current recommendations support RV vaccination in preterm infants and also in those with preexisting underlying disease, including gastrointestinal disease, in non-acute phases of illness [3,5,92]. Recently, it was shown that RV vaccination among short bowel patients is well tolerated [93].

Targeted RV vaccination does not offer the potential benefits of herd-protection, which has been described after implementation of universal RV vaccination. Observed effects among unvaccinated individuals ranged from 0 to 72% with substantial differences between consecutive years and effects declining with increasing age [46,58,59]. As severe RVGE occurs mainly in those <5 years old, herd-immunity could be a transient effect post-implementation, which disappears when coverage rates among this age-group approach 100%. Therefore, herd-immunity effects on the population level are difficult to predict [47]. Continued surveillance may provide more insights in coming years.

Naturally, our findings and conclusions may not hold for countries with high RV mortality among the general infant population and with higher RVGE incidences. In such countries universal RV vaccination remains the recommended approach.

## Conclusions

Universal RV vaccination is the preferred strategy to decrease the high disease burden among young children caused by RV in European countries and elsewhere, but is probably not cost-effective from the healthcare provider perspective. Targeted RV vaccination of high-risk infants is highly cost-effective and can nearly eliminate RV mortality in developed countries with very limited impact on healthcare budgets. We, therefore, encourage policy makers in countries without RV vaccination programs to prioritize RV vaccination for high-risk infants.

## Abbreviations

CI: confidence interval; GA: gestational age; GE: gastroenteritis; ICER: incremental cost-effectiveness ratio; LBW: low-birth weight; QALY: quality-adjusted life year; RR: risk ratio; RV: rotavirus; YLL: years of life lost.

## Competing interests

All authors declare they have no competing interests.

## Authors’ contributions

PB-V coordinated the study project. She was involved in study design and execution, data collection, analysis and interpretation and manuscript writing. MM was involved in data analysis and interpretation and assisted in drafting the manuscript. NH, MF, MvH, LW and WdW were involved in on-site study execution and data collection. NH, MF and MvH were also involved in data interpretation. MB supervised the study during all stages of the research process, was involved in data interpretation and assisted in drafting the manuscript. All authors have seen and approved the final version of the manuscript. PB-V and MM had full access to all of the data in the study and take responsibility for the integrity of the data and the accuracy of the data analysis.

## Pre-publication history

The pre-publication history for this paper can be accessed here:

http://www.biomedcentral.com/1741-7015/11/112/prepub

## Supplementary Material

Additional file 1 **Description nested case–control study: methods and results.** Additional tables with cost calculations and model results. Click here for file
