# Supplementary material for: Targeted rotavirus vaccination of high-risk infants; a low cost and highly cost-effective alternative to universal vaccination
Source: BMC Med. 2013 Apr 26;11:112. doi: 10.1186/1741-7015-11-112 (PMC3665442; doi:10.1186/1741-7015-11-112)
Supplement: Additional file 1 — Description nested case–control study: methods and results. Additional tables with cost calculations and model results. [file 1741-7015-11-112-S1.docx]

## Supplementary Material

### Observational Study; Nested Case Control study

A nested case-control study was performed within the observational study to determine whether children with prematurity, LBW or severe congenital pathology are at increased risk of acquiring nosocomial RVGE. All children hospitalized at the tertiary-care hospital and who acquired nosocomial RVGE during the 5 year study period were included as cases. Controls were randomly selected from the hospital population of the same hospital and were individually matched to the cases in a 1:2 ratio. Patients were eligible as controls when hospitalized for at least 72 hours and without a history of known previous RV infection. Matching on age and admission date (± 1 month) was performed to account for differences in age-dependent risk of RV disease and the seasonal circulation of RV. The eligible age-range for controls was ± 1 month difference for cases less than one year of age, ± 3 months difference for cases 1-2 year old and ± 6 months thereafter. Data extraction for cases and control occurred in a similar way by review of medical records.

### Statistical Analysis

Characteristics of cases and controls were compared using Chi-Square and Mann-Whitney U test. Case-control pairs were analyzed by using conditional logistic regression for binary outcomes to account for the matched design. We assessed the presence of potential risk factors prematurity, LBW and congenital pathology for nosocomial RVGE. Candidate covariates in the model were gender and admission ward. Results from conditional logistic regression analyses are reported as adjusted Odds Ratios (aOR) and 95% CIs.

### Results

Nested case-control study

One hundred and two nosocomial cases occurred at the tertiary care centre and were included in the case-control study. An age and admission date matched hospital control was available for 100 cases. Two suitable controls were available for 96 cases (Table S1). Presence of congenital pathology was less common among controls than among cases and length of stay was significantly shorter in controls. Patients with congenital pathology were significantly more likely to acquire RV infection during an episode of hospitalization than otherwise healthy children (aOR: 3.6, 95%CI: 1.8 – 7.0). Similar results were seen for children born before 36 weeks (aOR): 3.3, 95%CI: 1.5 – 7.3) or those with LBW ( aOR: 3.2, 95%CI: 1.5 – 7.1) The increased risk among patients with congenital pathology, prematurity and LBW is largely mediated through an increased length of stay as was shown by a sub-analysis in which only controls matched on exposure time were included (i.e. hospitalization of the control for at least the number of days until the case develops nosocomial RVGE). Although a smaller number of case-control pairs was included this analysis (N= 44 cases, 96 controls) the effect of all three risk factors was reduced and no longer significant (aOR congenital pathology: 1.4, 95%CI: 0.6 – 3.3; aOR GA<36weeks: 1.3, 95%CI: 0.4; 3.7; aOR LBW: 1.4, 95%CI: 0.5; 3.8). Irrespective of the underlying mechanism, due to the increased risk to acquire nosocomial RVGE during an episode of hospitalization, children with one of the risk factors are overrepresented among cases compared to controls.

Table S1. Standard Cost prices applied and units of resource use for RV hospitalizations*

| Hospital care RV hospitalizations^‡^ | | | | Mean units of resource used/  episode | | | | |  |
| --- | --- | --- | --- | --- | --- | --- | --- | --- | --- |
|  | |  |  | *NRV*  *Tertiary (N=103)* | *NRV General (N=73)* | *CARV Tertiary (N=157)* | *CARV General (N=603)* | | |
| Tertiary-care centre patient-day | € 595.95 [39] | |  | 3.01 | 0 | 5.50 | 0 | | |
| General hospital patient-day | € 450.85 [39] | |  | 0 | 2.57 | 0 | 3.65 | | |
| Intensive Care patient-day | € 2,262.55 [39] | |  | 0.34 | 0 | 0.35 | 0 | | |
| ER visit | € 156.90 [39] | |  | 0.12 | 0.08 | 0.94 | 0.98 | | |
| Additional costs for contact isolation | € 75.00 [41] | |  | 4.66 | 3.29 | 4.62 | 3.30 | | |
| Ambulance | € 522.14 [40] | |  | 0.05 | 0.00 | 0.03 | 0 | | |
| Standard cost prices^40^ of most frequent diagnostic and therapeutic interventions related to nosocomial RVGE and those with highest budget impact^†^ | | | | | | | |  |  |
| *Microbiology testing* |  | |  |  |  |  |  | | |
| RV immuno-essay | € 20.09 | |  | 0 | 0.44 |  |  | | |
| Combined rota-, adenovirus immuno-essay | € 40.18 | |  | 0.68 | 0.57 |  |  | | |
| RV viral culture | € 26.80 | |  | 0.64 | 0 |  |  | | |
| Norovirus PCR | € 202.21 | |  | 0.26 | 0.19 |  |  | | |
| Bacterial stool culture | € 40.19 | |  | 0.21 | 0.19 |  |  | | |
| C. difficile toxin | € 20.09 | |  | 0.09 | 0 |  |  | | |
| Bacterial blood culture | € 26.80 | |  | 0.23 | 0.09 |  |  | | |
| Urinary culture | € 21.43 | |  | 0.03 | 0.04 |  |  | | |
| *Blood chemistry/hematology* |  | |  |  |  |  |  | | |
| C-reactive Protein | € 4.69 | |  | 0.37 | 0.36 |  |  | | |
| Serum electrolytes (natrium, kalium, other) | € 3.40 | |  | 0.81 | 0.31 |  |  | | |
| Blood glucose | € 1.70 | |  | 0.37 | 0.15 |  |  | | |
| Blood Gas Analysis (pH, base excess, HCO3) | € 4.69 | |  | 0.34 | 0.18 |  |  | | |
| Renal function studies | € 3.40 | |  | 0.19 | 0.04 |  |  | | |
| Urinalysis | € 1.70 | |  | 0.06 | 0.08 |  |  | | |
| Complete blood cell (CBC) count | € 5.10 | |  | 0.38 | 0.35 |  |  | | |
| Differential count | € 3.35 | |  | 0.24 | 0.38 |  |  | | |
| *Imaging* |  | |  |  |  |  |  | | |
| Abdominal ultrasound | € 43.98 | |  | 0.11 | 0 |  |  | | |
| Abdominal X-ray | € 49.77 | |  | 0.11 | 0.04 |  |  | | |
| *Therapeutic interventions* |  | |  |  |  |  |  | | |
| Oral rehydration solution | € 1.92/ day | |  | €1.00 | €0.16 |  |  | | |
| Ringer's lactate | € 2.48/ day | |  | €0.80 | 0 |  |  | | |
| Insertion of Central venous catheter | € 348.60 | |  | 0.07 | 0 |  |  | | |
| Parental nutrition (depending on amount) | € 46-117/day | |  | €49.83 | €1.64 |  |  | | |
| Antimicrobial therapy (depending on agents and amount) | € 0.80-73.7/ day | |  | €12.15 | €0.91 |  |  | | |
| Other medication | List-price | |  | €6.24 | €1.76 |  |  | | |
| Prescription fee | € 9.49 | |  | 0.15 | 0.38 |  |  | | |
| Packed cells | € 208.24 | |  | 0.05 | 0 |  |  | | |
| Trombocytes | € 502.46 | |  | 0.03 | 0 |  |  | | |
| Fresh Frozen Plasma | € 179.23 | |  | 0.02 | 0 |  |  | | |

NRV: Nosocomial Rotavirus

CARV: Community-acquired Rotavirus

*Prices adjusted to 2011 level according to Dutch Consumer Price Index

‡Standard cost prices include staff, consumables, overheads, diagnostic tests and pharmaceuticals

†Costs for diagnostic and therapeutic interventions were only included for nosocomial RV patients when zero excess days of hospitalization were incurred

Table S2. Age Distribution of RV patients with different levels of healthcare and fatal cases

|  | | **0-2mo** | **2-4mo** | **4-6mo** | **6-12mo** | **0-1 yrs** | **1-2yrs** | **2-3yr** | **3-4yr** | **4-5yr** | **1-4yrs** | **5-9yr** | **10-14yr** | **Source** |
| --- | --- | --- | --- | --- | --- | --- | --- | --- | --- | --- | --- | --- | --- | --- |
| **RV incidence** | | 2% | 3% | 4% | 15% | 24% | 37% | 10% | 7% | 2% | 57% | 13% | 6% | Distributions for age groups <1, 1-4, 5-9 and 10-14 yr based on population cohort study, subdistributions for children < 1 yr and 1-4yr taken from RoHo study |
| **Hospitalizations** | |  |  |  |  |  |  |  |  |  |  |  |  |  |
| Ineligible | CA | 5% | 5% | 8% | 28% |  | 34% | 9% | 6% | 2% |  | 3% | 0% | RoHo study |
|  | Noso | 11% | 17% | 11% | 31% |  | 15% | 6% | 4% | 0% |  | 2% | 3% |  |
| Eligible | CA | 0% | 9% | 3% | 21% |  | 40% | 13% | 2% | 4% |  | 4% | 2% |  |
|  | Noso | 30% | 15% | 7% | 25% |  | 17% | 0% | 5% | 0% |  | 2% | 0% |  |
| **Mortality** |  |  |  |  |  |  |  |  |  |  |  |  |  |  |
| Ineligible |  | 14% | 28% | 0% | 42% |  | 14% | 0% | 0% | 0% |  | 0% | 0% | Assumed equal to mortality distribution among eligible |
| Eligible |  | 14% | 28% | 0% | 42% |  | 14% | 0% | 0% | 0% |  | 0% | 0% |  |

CA: Community-acquired

Noso: Nosocomial

Table S3. Characteristics of RV hospitalizations identified in the multi-centre observational study

|  | | **Community-acquired** | | **Nosocomial** | | **Total** | |
| --- | --- | --- | --- | --- | --- | --- | --- |
|  | | N=760 (81%) | | N=176 (19%) | | N=936 | |
| Hospital | |  |  |  |  |  |  |
|  | A* | 157 | (21%) | 103 | (59%) | 260 | (28%) |
|  | B | 157 | (21%) | 29 | (16%) | 186 | (20%) |
|  | C | 283 | (37%) | 35 | (20%) | 318 | (34%) |
|  | D | 163 | (21%) | 9 | (5%) | 172 | (18%) |
| Year† | |  |  |  |  |  |  |
|  | 2006 | 151 | (20%) | 24 | (14%) | 175 | (19%) |
|  | 2007 | 132 | (17%) | 31 | (18%) | 163 | (17%) |
|  | 2008 | 166 | (22%) | 28 | (16%) | 194 | (21%) |
|  | 2009 | 178 | (23%) | 56 | (32%) | 234 | (25%) |
|  | 2010 | 133 | (18%) | 37 | (21%) | 170 | (18%) |
| Male | | 413 | (54%) | 93 | (53%) | 506 | (54%) |
| Median Age (range) | | 13 mo | (3 days-18yrs) | 6 mo | (4 days-11yrs) | 12 mo | (0-18yrs) |
| Age < 15 weeks | | 65 | (8%) | 67 | (38%) | 132 | (14%) |
| GA < 36 weeks | | 43 | (6%) | 43 | (24%) | 83 | (9%) |
| Low Birth Weight (< 2500gr) | | 57 | (8%) | 47 | (27%) | 104 | (11%) |
| Presence of CCC | | 105 | (14%) | 114 | (65%) | 219 | (23%) |
|  | Congenital pathology | 56 | (8%) | 60 | (48%) | 116 | (15%) |

*Tertiary Care Hospital

†December 1st of the previous year until November 30st of the year stated

GA: Gestational Age

Hospital A: Wilhelmina Children’s Hospital, University Medical Centre Utrecht

Hospital B: Diakonessen Hospital, Utrecht

Hospital C: Spaarne Hospital, Hoofddorp

Hospital D: Kennemer Hospital, Haarlem

Table S4. Characteristics of nosocomial cases and controls

|  | | **Cases** |  | **Controls** |  | **P-value** |
| --- | --- | --- | --- | --- | --- | --- |
|  | | N=100 |  | N=196 |  |  |
| Age (median, range) | | 5.8 | (0-139) | 5.9 | (0-139) | 0.78 |
| Male (%) | | 54 | (54%) | 107 | (55%) | 0.92 |
| GA < 36 (%) | | 20 | (23%) | 33 | (24%) | 0.63 |
| LBW (%) | | 20 | (20%) | 34 | (17%) | 0.70 |
| Presence of CCC (%) | | 89 | (89%) | 119 | (61%) | <0.0001 |
|  | Congenital pathology (%) | 51 | (51%) | 61 | (31%) | 0.001 |
| Healthy† | | 10 | (10%) | 69 | (35%) | <0.0001 |
| Admission during RV season* (%) | | 63 | (63%) | 124 | (63%) | 0.96 |
| Ward type | |  |  |  |  |  |
|  | Pediatric medicine and surgery (%) | 60 | (60%) | 124 | (63%) | 0.19 |
|  | Hematology/ Oncology (%) | 17 | (17%) | 17 | (9%) |  |
|  | Neonatology (%) | 18 | (18%) | 43 | (22%) |  |
|  | Pediatric ICU (%) | 5 | (5%) | 12 | (6%) |  |
| Length of stay (median, range) | | 24.5 | (1-272) | 7.5 | (4-495) | <0.001 |

*January through April

†Gestational age ≥ 36 weeks, normal birth weight and no CCC.

Table S5. Results of cost-effectiveness analysis for universal and targeted RV vaccination in different scenarios (mean)

|  | | **Annual undiscounted Vaccination Costs (€ million)** | **Net Direct Healthcare Costs (€ million)** | **Net Societal Costs (€ million)** | **ICER, Healthcare perspective*** | **ICER, Societal perspective*** |
| --- | --- | --- | --- | --- | --- | --- |
| Universal RV vaccination | | |  |  |  |  |
| **RV1** | Basecase^&^ | 15,2 | 5,5 | 2,7 | 60,200 | 30,300 |
|  | Undiscounted | 15,2 | 7,1 | 3,4 | 42,100 | 20,700 |
|  | Discounted 5% | 15,2 | 4,7 | 2,4 | 71,400 | 36,700 |
|  | Discounted 4/1.5%† | 15,2 | 5,1 | 2,6 | 41,700 | 21,200 |
|  | Low vaccine price^&^ | 12,8 | 3,6 | 0,9 | 39,600 | 9,800 |
|  | High vaccine price^&^ | 17,7 | 7,4 | 4,6 | 80,500 | 50,700 |
|  | Herd immunity^&^ | 15,2 | 4,8 | 1,6 | 46,800 | 15,600 |
|  | Best case^&^ | 12,8 | 2,9 | -0,4 | 21,300 | cost-saving |
|  | Worst case^&^ | 17,4 | 7,4 | 4,7 | 82,900 | 53,300 |
|  | Free market Price^&^ | 25,0 | 13,0 | 10,3 | 141,400 | 111,700 |
| **RV5** | Basecase^&^ | 16,7 | 6,8 | 4,2 | 77,400 | 47,500 |
|  | Undiscounted | 16,7 | 8,8 | 5,3 | 53,800 | 32,500 |
|  | Discounted 5% | 16,7 | 5,2 | 2,5 | 71,300 | 35,100 |
|  | Discounted 4/1.5%† | 16,7 | 6,3 | 3,9 | 53,800 | 33,200 |
|  | Low vaccine price^&^ | 14,2 | 4,9 | 2,3 | 56,000 | 26,200 |
|  | High vaccine price^&^ | 19,1 | 8,7 | 6,0 | 98,500 | 68,600 |
|  | Herd immunity^&^ | 16,7 | 6,1 | 2,9 | 60,400 | 29,100 |
|  | Best case^&^ | 14,3 | 4,2 | 1,0 | 31,400 | 7,600 |
|  | Worst case^&^ | 18,8 | 8,7 | 6,1 | 102,100 | 72,200 |
|  | Free market Price^&^ | 26,5 | 14,3 | 11,7 | 162,600 | 132,600 |

|  | | **Annual undiscounted Vaccination Costs (€ million)** | **Net Direct Healthcare Costs**  **(€ million)** | **Net Societal Costs**  **(€ million)** | **ICER, Healthcare perspective*** | **ICER, Societal perspective*** |
| --- | --- | --- | --- | --- | --- | --- |
| Targeted RV vaccination | |  |  |  |  |  |
| **RV1** | Basecase^&^ | 1,5 | 0,1 | -0,2 | 2,600 | cost-saving |
|  | Undiscounted | 1,5 | 0,2 | -0,3 | 1,600 | cost-saving |
|  | Discounted 5% | 1,5 | 0,1 | -0,2 | 3,500 | cost-saving |
|  | Discounted 4/1.5%† | 1,5 | 0,1 | -0,2 | 1,800 | cost-saving |
|  | Low vaccine price^&^ | 1,2 | -0,1 | -0,4 | cost-saving | cost-saving |
|  | High vaccine price^&^ | 1,7 | 0,3 | 0,0 | 6,100 | 100 |
|  | Herd immunity^&^ | NA | NA | NA | NA | NA |
|  | Best case^&^ | 1,3 | -0,1 | -0,5 | cost-saving | cost-saving |
|  | Worst case^&^ | 1,3 | 0,3 | 0,0 | 5,400 | 700 |
|  | Free market Price^&^ | 1,9 | 0,5 | 0,1 | 8,700 | 2,700 |
| **RV5** | Basecase^&^ | 1,6 | 0,3 | 0,0 | 3,700 | cost-saving |
|  | Undiscounted | 1,6 | 0,3 | -0,2 | 2,800 | cost-saving |
|  | Discounted 5% | 1,6 | 0,2 | -0,1 | 5,800 | cost-saving |
|  | Discounted 4/1.5%† | 1,6 | 0,2 | -0,1 | 3,100 | cost-saving |
|  | Low vaccine price^&^ | 1,3 | 0,1 | -0,3 | 1,500 | cost-saving |
|  | High vaccine price^&^ | 1,8 | 0,4 | 0,1 | 8,100 | 2,100 |
|  | Herd immunity^&^ | NA | NA | NA | NA | NA |
|  | Best case^&^ | 1,3 | 0,0 | -0,3 | 900 | cost-saving |
|  | Worst case^&^ | 2,0 | 0,5 | 0,1 | 8,300 | 1,800 |
|  | Free market Price^&^ | 2,0 | 0,6 | 0,3 | 10,900 | 4,900 |

&Using a 3% discount rate for both, costs and effects

†According to Dutch guidelines for health economic evaluations: 4% discount rate for costs and 1·5% for benefits.

*Rounded to the last two integers

Figure S1

**
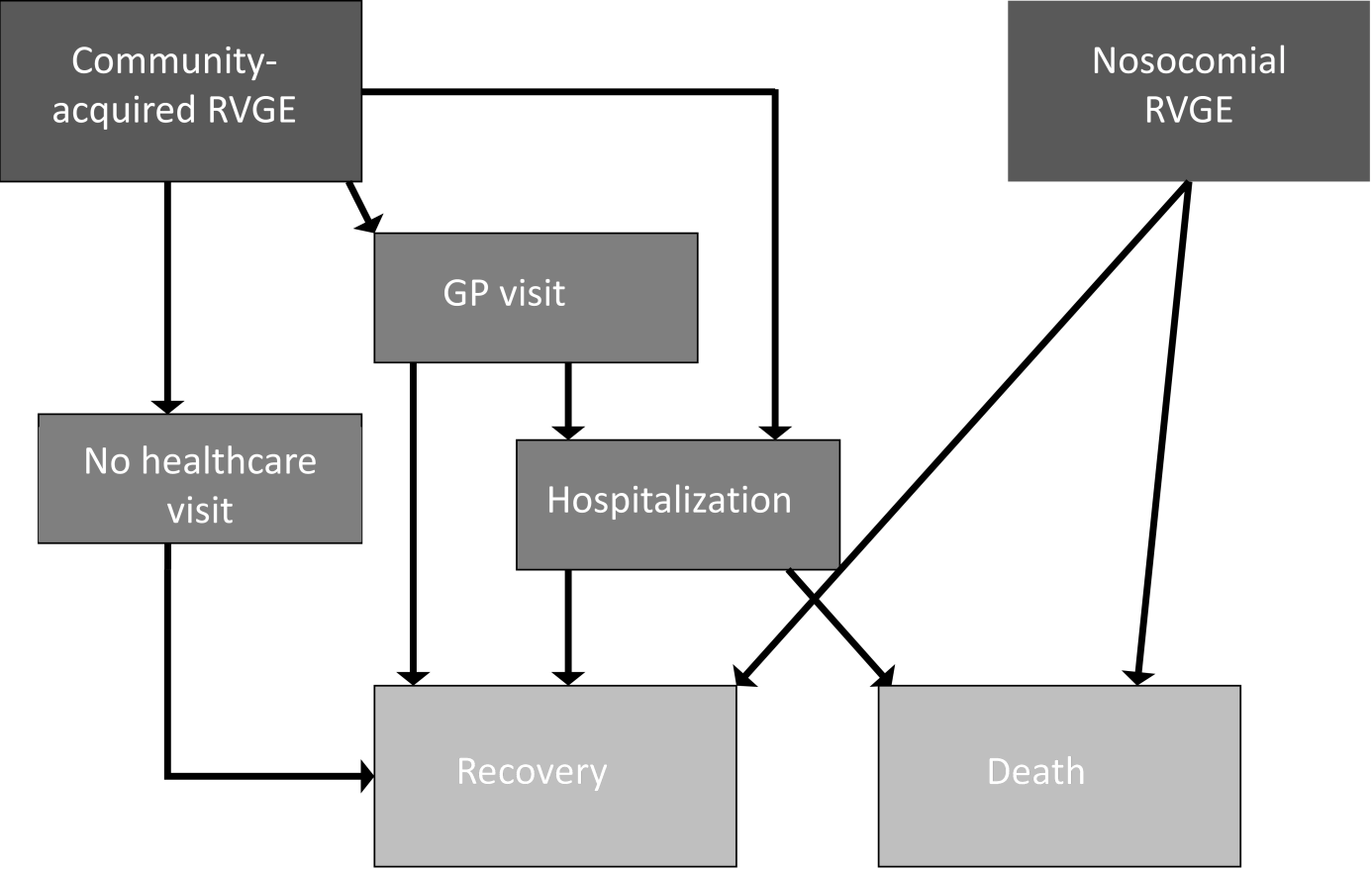
**

Figure S1: RV outcome tree and different healthcare paths considered in model.

Figure S2.

Figure S2: Cost-effectiveness plane showing results of 10,000 iterations for net discounted healthcare costs and QALY’s gained by either universal (red) or targeted (blue) RV vaccination compared to no vaccination under base-case assumptions and assuming 3% discount rate for both costs and effects. The black and dotted lines represent the €20,000 and €35,000/QALY thresholds for cost-effectiveness, respectively.
